# *N*′-(5-Chloro-2-hydroxy­benzyl­idene)-4-hydroxy­benzohydrazide

**DOI:** 10.1107/S160053680901215X

**Published:** 2009-04-08

**Authors:** Xiao-Yang Qiu

**Affiliations:** a Department of Chemistry, Shangqiu Normal University, Shangqiu 476000, People’s Republic of China

## Abstract

The title Schiff base compound, C_14_H_11_ClN_2_O_3_, was prepared by the reaction of 5-chloro­salicylaldehyde and 4-hydroxy­benzohydrazide. The mol­ecule exists in a *trans* configuration with respect to the methyl­idene group. The dihedral angle between the two benzene rings is 40.1 (2)°. An intra­molecular O—H⋯N hydrogen bond helps to stabilize the mol­ecular conformation. In the crystal structure, mol­ecules are linked into a three-dimensional network by inter­molecular N—H⋯O and O—H⋯O hydrogen bonds.

## Related literature

For the biological properties of hydrazone compounds, see: Bedia *et al.* (2006 ▶); Rollas *et al.* (2002 ▶); Fun *et al.* (2008 ▶). For the structures of hydrazone compounds we have reported previously, see: Qiu, Fang *et al.* (2006 ▶); Qiu, Luo *et al.*, (2006*a* ▶,*b* ▶); Qiu, Xu *et al.* (2006 ▶). For bond-length data, see: Allen *et al.* (1987 ▶). For related structures see: Singh *et al.* (2007 ▶); Narayana *et al.* (2007 ▶); Cui *et al.* (2007 ▶); Diao *et al.* (2008 ▶).
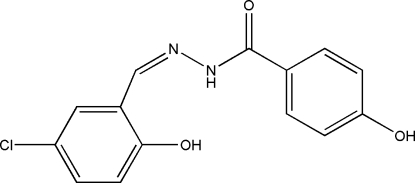


## Experimental

### 

#### Crystal data

C_14_H_11_ClN_2_O_3_
*M* *_r_* = 290.70
Orthorhombic, 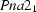

*a* = 9.423 (1) Å
*b* = 9.839 (1) Å
*c* = 13.770 (1) Å
*V* = 1276.7 (2) Å^3^
*Z* = 4
Mo *K*α radiation
μ = 0.31 mm^−1^
*T* = 298 K
0.17 × 0.15 × 0.15 mm

#### Data collection

Bruker SMART CCD diffractometer
Absorption correction: multi-scan (*SADABS*; Sheldrick, 1996 ▶) *T*_min_ = 0.950, *T*_max_ = 0.955
7398 measured reflections
2231 independent reflections
2144 reflections with *I* > 2σ(*I*)
*R*_int_ = 0.022

#### Refinement

*R*[*F* ^2^ > 2σ(*F* ^2^)] = 0.026
*wR*(*F* ^2^) = 0.071
*S* = 1.06
2231 reflections
187 parameters
2 restraints
H atoms treated by a mixture of independent and constrained refinement
Δρ_max_ = 0.21 e Å^−3^
Δρ_min_ = −0.32 e Å^−3^
Absolute structure: Flack (1983 ▶), 784 Friedel pairs
Flack parameter: −0.01 (6)

### 

Data collection: *SMART* (Bruker, 2001 ▶); cell refinement: *SAINT* (Bruker, 2001 ▶); data reduction: *SAINT*; program(s) used to solve structure: *SHELXS97* (Sheldrick, 2008 ▶); program(s) used to refine structure: *SHELXL97* (Sheldrick, 2008 ▶); molecular graphics: *SHELXTL* (Sheldrick, 2008 ▶); software used to prepare material for publication: *SHELXTL*.

## Supplementary Material

Crystal structure: contains datablocks I, global. DOI: 10.1107/S160053680901215X/sj2605sup1.cif

Structure factors: contains datablocks I. DOI: 10.1107/S160053680901215X/sj2605Isup2.hkl

Additional supplementary materials:  crystallographic information; 3D view; checkCIF report

## Figures and Tables

**Table 1:** Hydrogen-bond geometry (Å, °)

*D*—H⋯*A*	*D*—H	H⋯*A*	*D*⋯*A*	*D*—H⋯*A*
N2—H2⋯O2^i^	0.892 (10)	2.121 (11)	3.0065 (18)	172 (3)
O3—H3⋯O2^ii^	0.82	1.98	2.7479 (19)	157
O1—H1⋯N1	0.82	1.89	2.6057 (19)	145

Symmetry codes: (i) 
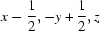
; (ii) 
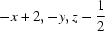
.

**Fig. 1.**
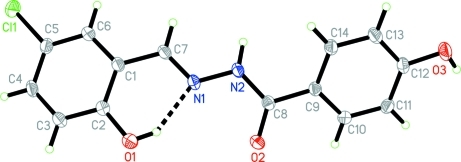
The molecular structure of (I), with atom labels and 30% probability displacement ellipsoids for non-H atoms.

**Fig. 2.**
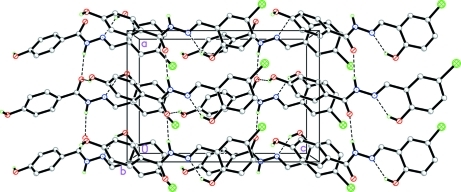
The crystal packing of (I), viewed along the b axis with hydrogen bonds drawn as dashed lines.

## supplementary crystallographic information

### Comment

Hyrazone compounds, derived from the reaction of aldehydes with hydrazides, have been widely studied due to their excellent biological properties (Bedia *et al.*, 2006; Rollas *et al.*, 2002; Fun *et al.*, 2008). Recently, we have reported several Schiff base hydrazone compounds (Qiu, Fang *et al.*, 2006; Qiu, Luo *et al.*, 2006*a*,b; Qiu, Xu *et al.*, 2006), and we report herein the crystal structure of the new title compound, (I), Fig. 1.

The molecule in (I) exists in a *trans* configuration with respect to the methylidene group. The dihedral angle between the two benzene rings is 40.1 (2)°. The bond lengths in (I) are found to have normal values (Allen *et al.*, 1987) and are comparable to the values found in similar compounds (Singh *et al.*, 2007; Narayana *et al.*, 2007; Cui *et al.*, 2007; Diao *et al.*, 2008).

An intramolecular O–H···N hydrogen bond (Table 1) helps to stabilize the molecular conformation. In the crystal structure, molecules are linked into a three-dimensional network by intermolecular N–H···O and O–H···O hydrogen bonds (Table 1 and Fig. 2).

### Experimental

The title compound was prepared by the Schiff base condensation of equimolar amounts (0.5 mmol each) of 5-chlorosalicylaldehyde and 4-hydroxybenzohydrazide in methanol (20 ml). Excess methanol was removed from the reaction mixture by distillation. The colourless solid was filtered and dried in air. Colourless block-shaped crystals suitable for X-ray diffraction were obtained from a methanol solution.

### Refinement

The imino H atoms were located in a difference map and refined with N–H distances restrained to 0.90 (1) Å. The remaining H atoms were positioned geometrically [C–H = 0.93 Å, O–H = 0.82 Å] and refined using a riding model, with *U*_iso_(H) = 1.2*U*_eq_(C) and 1.5*U*_eq_(O).

### Crystal data

**Table:**

C_14_H_11_ClN_2_O_3_	*F*(000) = 600
*M**_r_* = 290.70	*D*_x_ = 1.513 Mg m^−^^3^
Orthorhombic, *P**n**a*2_1_	Mo *K*α radiation, λ = 0.71073 Å
Hall symbol: P 2c -2n	Cell parameters from 4763 reflections
*a* = 9.423 (1) Å	θ = 2.5–30.6°
*b* = 9.839 (1) Å	µ = 0.31 mm^−^^1^
*c* = 13.770 (1) Å	*T* = 298 K
*V* = 1276.7 (2) Å^3^	Block, colourless
*Z* = 4	0.17 × 0.15 × 0.15 mm

### Data collection

**Table:**

Bruker SMART CCD diffractometer	2231 independent reflections
Radiation source: fine-focus sealed tube	2144 reflections with *I* > 2σ(*I*)
graphite	*R*_int_ = 0.022
ω scans	θ_max_ = 27.0°, θ_min_ = 2.5°
Absorption correction: multi-scan (*SADABS*; Sheldrick, 1996)	*h* = −12→11
*T*_min_ = 0.950, *T*_max_ = 0.955	*k* = −12→12
7398 measured reflections	*l* = −17→11

### Refinement

**Table:**

Refinement on *F*^2^	Hydrogen site location: inferred from neighbouring sites
Least-squares matrix: full	H atoms treated by a mixture of independent and constrained refinement
*R*[*F*^2^ > 2σ(*F*^2^)] = 0.026	*w* = 1/[σ^2^(*F*_o_^2^) + (0.0409*P*)^2^ + 0.1827*P*] where *P* = (*F*_o_^2^ + 2*F*_c_^2^)/3
*wR*(*F*^2^) = 0.071	(Δ/σ)_max_ = 0.001
*S* = 1.06	Δρ_max_ = 0.21 e Å^−^^3^
2231 reflections	Δρ_min_ = −0.32 e Å^−^^3^
187 parameters	Extinction correction: *SHELXL97* (Sheldrick, 2008), Fc^*^=kFc[1+0.001xFc^2^λ^3^/sin(2θ)]^-1/4^
2 restraints	Extinction coefficient: 0.034 (3)
Primary atom site location: structure-invariant direct methods	Absolute structure: Flack (1983), 784 Friedel pairs
Secondary atom site location: difference Fourier map	Flack parameter: −0.01 (6)

### Special details

**Table:**

Geometry. All e.s.d.'s (except the e.s.d. in the dihedral angle between two l.s. planes) are estimated using the full covariance matrix. The cell e.s.d.'s are taken into account individually in the estimation of e.s.d.'s in distances, angles and torsion angles; correlations between e.s.d.'s in cell parameters are only used when they are defined by crystal symmetry. An approximate (isotropic) treatment of cell e.s.d.'s is used for estimating e.s.d.'s involving l.s. planes.
Refinement. Refinement of *F*^2^ against ALL reflections. The weighted *R*-factor *wR* and goodness of fit *S* are based on *F*^2^, conventional *R*-factors *R* are based on *F*, with *F* set to zero for negative *F*^2^. The threshold expression of *F*^2^ > σ(*F*^2^) is used only for calculating *R*-factors(gt) *etc*. and is not relevant to the choice of reflections for refinement. *R*-factors based on *F*^2^ are statistically about twice as large as those based on *F*, and *R*- factors based on ALL data will be even larger.

### Fractional atomic coordinates and isotropic or equivalent isotropic displacement parameters (Å^2^)

**Table:**

	*x*	*y*	*z*	*U*_iso_*/*U*_eq_	
Cl1	0.74914 (6)	0.47741 (5)	1.21657 (5)	0.05224 (15)	
N1	0.99352 (15)	0.30628 (14)	0.80967 (10)	0.0316 (3)	
N2	0.94949 (14)	0.28112 (13)	0.71621 (12)	0.0319 (3)	
O1	1.19292 (14)	0.32175 (17)	0.94031 (11)	0.0504 (4)	
H1	1.1598	0.3057	0.8865	0.076*	
O2	1.14143 (12)	0.14658 (12)	0.69247 (10)	0.0370 (3)	
O3	0.84616 (17)	0.10538 (15)	0.27816 (10)	0.0505 (4)	
H3	0.8733	0.0312	0.2583	0.076*	
C1	0.94953 (17)	0.38640 (15)	0.96863 (13)	0.0312 (3)	
C2	1.08639 (18)	0.35624 (17)	1.00215 (14)	0.0346 (4)	
C3	1.1165 (2)	0.36019 (19)	1.10078 (15)	0.0403 (4)	
H3A	1.2072	0.3386	1.1225	0.048*	
C4	1.0133 (2)	0.39569 (17)	1.16663 (14)	0.0391 (4)	
H4	1.0332	0.3960	1.2328	0.047*	
C5	0.87956 (19)	0.43088 (17)	1.13363 (14)	0.0362 (4)	
C6	0.84744 (18)	0.42738 (18)	1.03619 (14)	0.0352 (4)	
H6	0.7573	0.4524	1.0152	0.042*	
C7	0.90835 (18)	0.36642 (16)	0.86788 (13)	0.0325 (3)	
H7	0.8207	0.3972	0.8459	0.039*	
C8	1.03211 (17)	0.20101 (16)	0.65983 (13)	0.0292 (3)	
C9	0.98366 (16)	0.17900 (16)	0.55911 (12)	0.0296 (3)	
C10	1.03520 (17)	0.06631 (17)	0.50845 (14)	0.0335 (4)	
H10	1.0998	0.0084	0.5384	0.040*	
C11	0.99177 (18)	0.03955 (17)	0.41486 (14)	0.0350 (4)	
H11	1.0265	−0.0361	0.3821	0.042*	
C12	0.89604 (19)	0.12597 (18)	0.36973 (13)	0.0352 (4)	
C13	0.8457 (2)	0.2394 (2)	0.41833 (15)	0.0450 (5)	
H13	0.7826	0.2980	0.3876	0.054*	
C14	0.8889 (2)	0.26545 (18)	0.51195 (14)	0.0392 (4)	
H14	0.8544	0.3417	0.5441	0.047*	
H2	0.8598 (14)	0.303 (3)	0.703 (2)	0.080*	

### Atomic displacement parameters (Å^2^)

**Table:**

	*U*^11^	*U*^22^	*U*^33^	*U*^12^	*U*^13^	*U*^23^
Cl1	0.0578 (3)	0.0676 (3)	0.0313 (2)	0.0148 (2)	0.0054 (2)	−0.0062 (3)
N1	0.0343 (7)	0.0371 (7)	0.0235 (8)	−0.0016 (5)	−0.0046 (6)	−0.0003 (6)
N2	0.0319 (7)	0.0408 (7)	0.0229 (7)	0.0001 (5)	−0.0045 (7)	−0.0009 (6)
O1	0.0365 (7)	0.0791 (10)	0.0354 (8)	0.0131 (7)	−0.0033 (6)	−0.0060 (7)
O2	0.0323 (6)	0.0466 (6)	0.0322 (7)	0.0039 (5)	−0.0061 (5)	0.0025 (5)
O3	0.0679 (10)	0.0545 (8)	0.0289 (8)	0.0089 (6)	−0.0118 (7)	−0.0097 (6)
C1	0.0344 (8)	0.0322 (8)	0.0271 (9)	−0.0001 (6)	−0.0049 (7)	−0.0006 (6)
C2	0.0352 (8)	0.0376 (8)	0.0311 (9)	0.0014 (6)	−0.0033 (8)	−0.0024 (7)
C3	0.0400 (10)	0.0450 (9)	0.0358 (11)	0.0036 (7)	−0.0127 (8)	−0.0025 (8)
C4	0.0531 (11)	0.0398 (8)	0.0244 (9)	0.0025 (7)	−0.0103 (8)	−0.0032 (7)
C5	0.0444 (9)	0.0359 (8)	0.0283 (9)	0.0031 (7)	0.0005 (7)	−0.0044 (7)
C6	0.0350 (8)	0.0406 (8)	0.0300 (9)	0.0035 (7)	−0.0046 (7)	−0.0008 (7)
C7	0.0317 (8)	0.0386 (8)	0.0271 (9)	0.0007 (6)	−0.0056 (7)	0.0011 (7)
C8	0.0298 (8)	0.0318 (7)	0.0261 (9)	−0.0043 (6)	−0.0007 (6)	0.0027 (6)
C9	0.0304 (7)	0.0342 (7)	0.0243 (9)	−0.0013 (6)	0.0003 (6)	0.0019 (6)
C10	0.0309 (8)	0.0371 (8)	0.0327 (10)	0.0039 (6)	−0.0011 (7)	−0.0011 (7)
C11	0.0358 (9)	0.0370 (8)	0.0321 (10)	0.0015 (6)	0.0030 (7)	−0.0058 (7)
C12	0.0392 (9)	0.0426 (9)	0.0239 (9)	−0.0033 (7)	−0.0011 (7)	−0.0004 (7)
C13	0.0593 (12)	0.0446 (9)	0.0311 (10)	0.0158 (8)	−0.0110 (9)	0.0004 (8)
C14	0.0527 (10)	0.0360 (8)	0.0290 (9)	0.0111 (7)	−0.0052 (8)	−0.0040 (7)

### Geometric parameters (Å, °)

**Table:**

Cl1—C5	1.7391 (19)	C4—C5	1.384 (3)
N1—C7	1.279 (2)	C4—H4	0.9300
N1—N2	1.375 (2)	C5—C6	1.376 (3)
N2—C8	1.353 (2)	C6—H6	0.9300
N2—H2	0.892 (10)	C7—H7	0.9300
O1—C2	1.359 (2)	C8—C9	1.476 (2)
O1—H1	0.8200	C9—C14	1.394 (2)
O2—C8	1.245 (2)	C9—C10	1.397 (2)
O3—C12	1.361 (2)	C10—C11	1.378 (3)
O3—H3	0.8200	C10—H10	0.9300
C1—C6	1.398 (2)	C11—C12	1.387 (2)
C1—C2	1.402 (2)	C11—H11	0.9300
C1—C7	1.454 (2)	C12—C13	1.385 (3)
C2—C3	1.388 (3)	C13—C14	1.376 (3)
C3—C4	1.374 (3)	C13—H13	0.9300
C3—H3A	0.9300	C14—H14	0.9300
			
C7—N1—N2	118.72 (14)	N1—C7—C1	119.54 (15)
C8—N2—N1	117.94 (13)	N1—C7—H7	120.2
C8—N2—H2	125 (2)	C1—C7—H7	120.2
N1—N2—H2	116 (2)	O2—C8—N2	121.31 (16)
C2—O1—H1	109.5	O2—C8—C9	122.14 (15)
C12—O3—H3	109.5	N2—C8—C9	116.53 (14)
C6—C1—C2	118.37 (16)	C14—C9—C10	118.32 (16)
C6—C1—C7	119.37 (15)	C14—C9—C8	123.12 (15)
C2—C1—C7	122.09 (16)	C10—C9—C8	118.56 (14)
O1—C2—C3	117.99 (16)	C11—C10—C9	121.03 (16)
O1—C2—C1	121.73 (17)	C11—C10—H10	119.5
C3—C2—C1	120.28 (16)	C9—C10—H10	119.5
C4—C3—C2	120.55 (16)	C10—C11—C12	119.69 (16)
C4—C3—H3A	119.7	C10—C11—H11	120.2
C2—C3—H3A	119.7	C12—C11—H11	120.2
C3—C4—C5	119.41 (17)	O3—C12—C13	116.71 (16)
C3—C4—H4	120.3	O3—C12—C11	123.28 (16)
C5—C4—H4	120.3	C13—C12—C11	120.01 (17)
C6—C5—C4	120.95 (18)	C14—C13—C12	120.13 (17)
C6—C5—Cl1	119.44 (14)	C14—C13—H13	119.9
C4—C5—Cl1	119.59 (15)	C12—C13—H13	119.9
C5—C6—C1	120.32 (16)	C13—C14—C9	120.80 (16)
C5—C6—H6	119.8	C13—C14—H14	119.6
C1—C6—H6	119.8	C9—C14—H14	119.6

### Hydrogen-bond geometry (Å, °)

**Table:**

*D*—H···*A*	*D*—H	H···*A*	*D*···*A*	*D*—H···*A*
N2—H2···O2^i^	0.89 (1)	2.12 (1)	3.0065 (18)	172 (3)
O3—H3···O2^ii^	0.82	1.98	2.7479 (19)	157
O1—H1···N1	0.82	1.89	2.6057 (19)	145

Symmetry codes: (i) *x*−1/2, −*y*+1/2, *z*; (ii) −*x*+2, −*y*, *z*−1/2.
